# 915. Prevalence and Treatment of Genital Herpes in a United States Administrative Claims Database

**DOI:** 10.1093/ofid/ofad500.960

**Published:** 2023-11-27

**Authors:** Emma Viscidi, Purva Jain, Alan Embry, Brent Arakaki, Irisdaly Estevez, Zachary Marcum

**Affiliations:** Moderna Therapeutics, Cambridge, Massachusetts; Aetion, Inc., New York City, New York; Moderna Therapeutics, Cambridge, Massachusetts; Aetion, Inc., New York City, New York; Aetion, Inc., New York City, New York; Aetion, Inc., New York City, New York

## Abstract

**Background:**

Herpes simplex virus type-2 (HSV-2) is the most frequent cause of genital herpes (GH) in the United States (US). There is no cure for HSV-2, but antiviral drugs can reduce outbreaks. Limited epidemiologic data exist on the contemporary burden of GH and treatment. The objective of this study was to estimate the annual GH prevalence and antiviral treatment rates.

**Methods:**

This observational study utilized cohorts to calculate annual period prevalence rates of GH and included US administrative claims data from HealthVerity medical and pharmacy claims of continuously enrolled insured individuals. The crude and age-sex standardized prevalence rates of GH and recurrent GH were calculated from 01/01/2019 to 12/31/2021, by calendar year. GH was defined as >1 ICD-10 diagnosis code. Recurrent GH was defined as >2 GH diagnosis codes, at least 28 days apart, or >1 GH diagnosis code followed by at least 2 non-overlapping antiviral drug claims. Among those with GH, the proportion of patients untreated or on episodic or suppressive antiviral treatment was estimated. Episodic treatment was defined as <6 months of antiviral treatment, and suppressive treatment was defined >6 months of antiviral treatment, within each calendar year. Analyses were stratified by age, sex, and immunocompromised status.

**Results:**

From 2019 to 2021, the age-sex standardized prevalence rates of GH ranged from 236 to 280 per 100,000 person-years (py) and from 81 to 98 per 100,000 py for recurrent GH. The prevalence rates of GH and recurrent GH were highest among females, those 25-29 years of age, and in immunocompromised individuals. Approximately one-third of patients with GH were untreated (32%-35%) in a given year. Among those treated for GH, the majority received episodic antiviral treatment (80%) rather than suppressive treatment (20%).

**Conclusion:**

GH prevalence rates were 236 to 280 per 100,000 py and recurrent GH was one-third of this. GH and recurrent GH were more common in females, young adults, and immunocompromised individuals. Approximately two-thirds of GH patients were treated with antivirals, and the majority of these received episodic treatment.

**Disclosures:**

**Emma Viscidi, PhD, MHS**, Moderna: Employee|Moderna: Stocks/Bonds **Alan Embry, PhD**, Moderna Therapeutics: Stocks/Bonds **Brent Arakaki, BS**, Aetion, Inc.: Employee

